# Systematic Review: Exploring Inter-Species Variability in Diabetes Mellitus for Translational Medicine

**DOI:** 10.3390/life16010064

**Published:** 2025-12-31

**Authors:** Luminița Diana Hrițcu, Vasile Boghian, Geta Pavel, Teodor Daniel Hrițcu, Florin Nechifor, Alexandru Spataru, Alexandra Andreea Cherșunaru, Alexandru Munteanu, Manuela Ciocoiu, Mihaela-Claudia Spataru

**Affiliations:** 1 Faculty of Veterinary Medicine, Ion Ionescu de la Brad Iasi University of Life Science, 700489 Iasi, Romania; luminita.hritcu@iuls.ro (L.D.H.); vasile.boghian@iuls.ro (V.B.);; 2 Faculty of Medicine, Grigore T. Popa University of Medicine and Pharmacy of Iasi, 700115 Iasi, Romaniamanuela.ciocoiu@umfiasi.ro (M.C.)

**Keywords:** animal models, metabolic disease, β-cell dysfunction, comparative medicine, gut microbiota, One Health

## Abstract

Interspecies variability in diabetes mellitus (DM) represents a critical challenge for translational medicine, as metabolic pathways, pancreatic architecture, and therapeutic responses differ substantially across animal models. This systematic review, conducted according to PRISMA 2020 guidelines, synthesized evidence from 86 eligible studies published between 2001 and 2025. Comparative data from rodents, dogs, cats, pigs, non-human primates, and humans were analyzed to identify species-specific patterns in insulin secretion, insulin resistance (IR), β-cell dysfunction, microbiota–metabolism interactions, and susceptibility to diabetic complications. Results indicate that spontaneous diabetes in dogs closely mirrors human type 1 diabetes (T1DM), whereas feline obesity-associated diabetes reflects key features of human type 2 diabetes (T2DM). Rodent models remain essential for mechanistic and genetic studies but show limited chronicity and lower predictive fidelity for long-term outcomes. Non-human primates exhibit the highest physiological similarity to humans, especially regarding β-cell structure and incretin response, supporting their role in advanced translational studies. Major limitations included methodological heterogeneity and inconsistent molecular reporting. Integrating spontaneous models with standardized protocols and multi-omics approaches enhances translational relevance and supports more accurate model selection in diabetes research.

## 1. Introduction

DM is a heterogeneous metabolic disorder characterized by chronic hyperglycemia caused by impaired insulin secretion, defective insulin action, or a combination of both [1,2,3,4,5]. Its global prevalence continues to rise due to the interplay of genetic factors, environmental exposures, dietary patterns, and immunometabolic dysregulation [6,7,8]. Despite major advances in clinical and experimental endocrinology, the mechanisms underlying the onset, progression, and heterogeneity of DM remain only partially understood across species.

Translational medicine aims to accelerate the transfer of mechanistic discoveries into effective diagnostic and therapeutic applications, thereby improving clinical outcomes [9,10,11,12,13,14]. In the context of diabetes mellitus, translational relevance depends critically on the appropriate selection and interpretation of experimental and spontaneous animal models, as species-specific differences directly influence the predictive value of preclinical findings for clinical decision-making and therapeutic development. Within this framework, animal models play a central role in elucidating β-cell physiology, insulin signaling pathways, inflammatory mechanisms, and metabolic homeostasis [15,16,17]. However, substantial differences in pancreatic architecture, immune regulation, metabolic rate, microbiota composition, and susceptibility to complications result in heterogeneous translational relevance among species [18,19,20,21,22,23].

Rodent models are widely used due to their genetic accessibility, standardized husbandry, and cost-effectiveness. Nevertheless, their ability to replicate the chronic evolution and phenotypic complexity of human DM is limited, particularly with respect to complications such as nephropathy, neuropathy, and retinopathy [21,22,23]. In contrast, companion animals frequently develop spontaneous forms of DM that closely parallel human disease. Dogs exhibit immune-mediated β-cell destruction and insulin dependence characteristic of human T1DM [24,25,26,27], whereas cats develop obesity-associated insulin resistance (IR) and progressive β-cell dysfunction that resembles human T2DM [24,26,27,28].

Despite these advantages, the translational success rate of preclinical diabetes research remains modest. Contributing factors include species-specific metabolic divergence, variability in islet cytoarchitecture, differences in mitochondrial efficiency, incretin response, gut microbiota–metabolism interactions, and inflammatory signaling [16,17,21,29,30]. Methodological heterogeneity—such as inconsistent diagnostic criteria, differences in induction methods, and limited molecular characterization—further reduces reproducibility and complicates cross-species comparisons [16,31].

Genetic factors also contribute to species-specific patterns of susceptibility. For example, polymorphisms in transcription factor 7-like 2 (TCF7L2), potassium inwardly rectifying channel subfamily J member 11 (KCNJ11), and peroxisome proliferator-activated receptor gamma (PPARG) influence metabolic risk and treatment response in humans [32]. Similarly, well-documented breed predispositions in dogs (e.g., Samoyeds, Miniature Schnauzers, Poodles) and hereditary β-cell dysfunction in Burmese cats highlight the importance of comparative genetic variability in translational research [24,26,27,29]. The One Health (OH) framework provides an integrative lens for studying metabolic diseases shared by humans and animals, emphasizing the influence of environmental exposures, diet, lifestyle, and microbiota composition on metabolic outcomes. Companion animals share many environmental determinants with their owners, making them valuable sentinel species for understanding lifestyle-associated metabolic dysregulation.

Given these challenges and opportunities, a comprehensive synthesis of interspecies differences in DM is essential to refine experimental design, guide appropriate model selection, increase reproducibility, and strengthen translational pathways from preclinical discovery to clinical application [14,15,16,17,18,19,20,22].

This systematic review was conducted in accordance with PRISMA 2020 guidelines [33] to summarize comparative evidence on DM across species, focusing on pathophysiological mechanisms, metabolic features, biomarker variability, and therapeutic implications.

Despite the growing number of reviews addressing DM in individual species or focusing on specific experimental models, a comprehensive synthesis that systematically compares interspecies variability within a translational medicine framework remains limited. Most existing reviews emphasize either mechanistic insights derived from rodent models or clinical aspects of human diabetes, with relatively little integration of spontaneous disease models in companion animals or cross-species translational relevance. The present systematic review addresses this gap by providing a structured, comparative analysis of DM across species, integrating experimental, clinical, and OH perspectives to better inform translational applicability.

## 2. Materials and Methods

**Figure 1:**
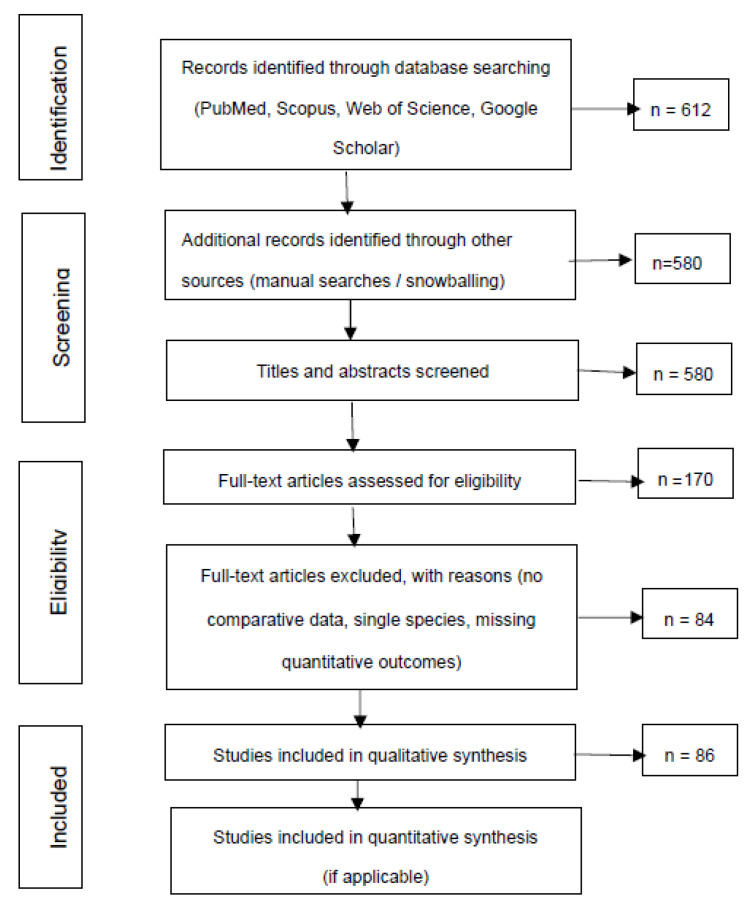
PRSIMA flow diagram of the selection process of studies included in the systematic review.

This systematic review was conducted according to the PRISMA 2020 guidelines [33] The PRISMA checklist and the flow diagram are provided in the Supplementary Material and Figure 1. No review protocol was registered in a public database (e.g., PROSPERO), and no pre-specified protocol was prepared prior to study initiation. The objective was to identify, appraise, and synthesize comparative evidence regarding interspecies variability in the pathophysiology, metabolic mechanisms, and treatment responses of diabetes mellitus (DM).

### 2.1. Literature Search Strategy

A comprehensive search was performed in PubMed, Scopus, Web of Science, and Google Scholar for studies published between 2001 and 2025. Search terms included combinations of keywords and MeSH/EmTree entries such as: “diabetes mellitus,” “insulin resistance,” “pancreatic β-cell function,” “interspecies variability,” “animal model,” “translational medicine,” “mouse,” “rat,” “dog,” “canine,” “cat,” “feline,” “non-human primate,” and “human.” Only articles published in English were considered to ensure consistency in methodological reporting. Additional references were identified through manual screening of bibliographies (snowballing).

### 2.2. Eligibility Criteria

Studies were included if they met all of the following criteria, defined a priori according to PRISMA 2020 recommendations:

(1) original experimental or observational studies investigating DM in animal models (rodents, dogs, cats, pigs, or non-human primates), with clearly described objectives and methodology;
(2) studies evaluating pathophysiological, molecular, metabolic, microbiome-related, or therapeutic aspects relevant to translational medicine;
(3) studies reporting quantitative or qualitative outcomes related to glucose homeostasis, insulin secretion, IR, β-cell function, or treatment response;
(4) full-text peer-reviewed publications available in English.

Exclusion criteria were: (1) reviews, editorials, commentaries, or conference abstracts; (2) studies limited to a single species without comparative relevance; (3) studies lacking original data or presenting insufficient methodological detail.

### 2.3. Study Selection

All titles and abstracts were screened independently by two reviewers. Full-text evaluation was conducted for potentially eligible articles, and disagreements were resolved by consensus. A total of 86 studies met all inclusion criteria and were included in the qualitative synthesis.

### 2.4. Data Extraction and Synthesis

For each included study, the following information was extracted: species examined, DM type (T1DM; T2DM), induction or disease model, study design, diagnostic criteria, outcome measures, and reported translational implications. Additionally, microbiome-related data were extracted from 27 eligible studies. Dominant bacterial genera were recorded and summarized as relative frequencies, representing the proportion of studies reporting each genus as dominant.

Due to heterogeneity in study designs, species, induction methods, and outcome measures, a narrative synthesis approach was applied. Comparative interpretations were supported by descriptive tables and figures illustrating interspecies differences in metabolic mechanisms, insulin signaling, and therapeutic response.

### 2.5. Quality Assessment and Risk of Bias

The methodological quality of included studies was evaluated based on comparative design, clarity of reporting, and methodological rigor. Risk of bias was assessed qualitatively, considering randomization, blinding, sample size, outcome reporting, and external validity. Publication bias was addressed descriptively due to the heterogeneity of study types and outcomes. The study selection process is summarized in the PRISMA 2020 flow diagram (Figure 1).

Figure 1 summarizes the identification, screening, eligibility, and inclusion steps following PRISMA 2020 guidelines. It presents the number of records retrieved, duplicates removed, screening criteria, full-text assessments, and the final 86 studies included in the qualitative synthesis, providing transparency in the study selection process.

## 3. Results and Discussions

### 3.1. Translational Relevance of DM Across Species

Companion and experimental animal models exhibit varying degrees of physiological and pathophysiological similarity to human DM, which directly influences their translational value. Canine models remain particularly relevant due to their spontaneous development of immune-mediated β-cell destruction, chronic hyperglycemia, and insulin dependence—hallmarks of human T1DM [15,27,31,34]. Their immune–metabolic responses, pancreatic architecture, and progression of β-cell dysfunction allow meaningful comparison with human disease and support their use in studies on islet transplantation and cellular therapies.

Rodent models, including chemically induced or genetically modified strains, contribute essential mechanistic insights into glucose–insulin dynamics, β-cell loss, and molecular signaling pathways [18,32,34]. However, marked interspecies differences in islet cytoarchitecture, immune responses, mitochondrial function, and cardiometabolic regulation limit their ability to reproduce the chronicity and complication patterns of human DM [21,34,35]. Consequently, rodent findings require cautious interpretation when extrapolated to clinical settings.

Comparative transcriptomic and co-expression analyses have highlighted conserved molecular signatures across species, enabling improved calibration of experimental models and informing species selection for targeted therapeutic studies [17,35]. Nonetheless, reproducibility challenges—stemming from methodological heterogeneity, small sample sizes, and limited standardization across laboratories—remain significant barriers to translational predictability [16,32,34].

Table 1 provides a comparative overview of major diabetes models across species, highlighting the type of diabetes modeled, key pathophysiological features, translational relevance, and principal limitations [15,33,34,36,37,38].

Animal models of DM also display substantial intra-species variability depending on the induction method and underlying pathophysiology. In rodents, DM is commonly induced chemically—through STZ- or alloxan-mediated β-cell destruction—or genetically, such as in non-obese diabetic (NOD) mice for autoimmune T1DM, and db/db or ob/ob mice for obesity-related T2DM [15,22,27,31,39]. While these models allow controlled experimental manipulation, they do not replicate the chronic, multifactorial progression observed in human DM.

Canine models include both spontaneous autoimmune diabetes, closely mirroring human T1DM, and chemically or surgically induced variants used primarily for mechanistic and transplantation research. The spontaneous canine form, characterized by complete insulin dependence and immune-mediated β-cell loss, provides superior translational value compared with induced models [15,22]. Similarly, feline models include spontaneous obesity-associated T2DM—which reflects human metabolic syndrome, IR, and progressive β-cell dysfunction—as well as induced models generated through high-fat diets or glucocorticoid administration. The spontaneous feline form demonstrates progressive β-cell failure and reversible glucose intolerance, features directly relevant to early human T2DM and prediabetes [24,26,27,29].

Distinguishing these induction mechanisms is essential for accurate cross-species comparisons, as each model type captures different aspects of disease mechanisms, therapeutic responses, and translational predictability. Spontaneous, chemically induced, genetic, and surgical models represent distinct biological constructs rather than variations in the same disease, and therefore require tailored interpretation [15,22,27,31,38].

Critical appraisal of the selected studies revealed considerable heterogeneity in design, reporting quality, and external validity. Clinical studies in dogs often applied rigorous methodologies—including histopathologic evaluation, immunologic profiling, and longitudinal monitoring—but were limited by small sample sizes and genetic variability, which increase uncertainty in translational estimates [15,22]. Rodent studies benefit from genetic control and large datasets but remain constrained by species-specific limitations in islet architecture and immune function, reducing predictive fidelity [15,16].

Risk-of-bias assessments frequently identified insufficient randomization, inconsistent use of control groups, and selective outcome reporting as major methodological concerns [11,30]. Certainty of evidence for translational outcomes—such as islet transplantation success or post-therapy glycemic improvement—ranged from moderate to low depending on species and study quality [34,39].

**Figure 2:**
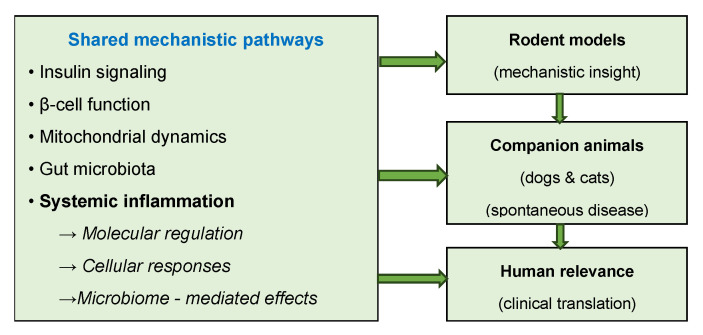
Translational and mechanistic framework of diabetes models across species.

To improve reproducibility and model comparability, standardized registries, minimal reporting frameworks, and data-sharing initiatives are required, enabling robust meta-analyses and cross-species calibration of predictive algorithms [11,34]. Figure 2 provides a graphical summary of these translational considerations, illustrating the progression from experimental control in animal models to the complex heterogeneity of human DM.

Bridging this gap requires a tiered translational strategy in which mechanistic hypotheses are first explored using rodent models and subsequently validated in spontaneous canine or feline DM. Harmonized protocols, shared biobanks, and standardized metabolic and molecular endpoints would support reproducible interspecies comparisons. Ethical refinement—through non-invasive monitoring, data reuse, and computational modeling—can reduce animal use while increasing predictive accuracy. OH collaborations integrating veterinary and human research centers may further strengthen data interoperability and accelerate translational progress across species.

Figure 2 summarizes a conceptual framework linking shared mechanistic pathways to increasing translational relevance across species.

### 3.2. Molecular and Cellular Mechanisms Underlying Interspecies Variability

Comparative analysis of the included studies revealed distinct molecular and cellular mechanisms underlying DM across species. Dogs predominantly exhibit insulin-deficient DM associated with immune-mediated β-cell dysfunction, closely paralleling human T1DM, whereas cats frequently develop obesity-associated IR accompanied by secondary β-cell failure, resembling early-stage human T2DM [23,24,29]. These differences are essential for interpreting interspecies variability in insulin signaling and metabolic regulation.

As conceptually summarized in Figure 2, molecular and cellular mechanisms underlying interspecies variability in DM include alterations in insulin signaling, β-cell function, mitochondrial dynamics, gut microbiota interactions, and systemic inflammation.

The included studies investigated multiple mechanistic domains, including insulin signaling pathways, β-cell function, mitochondrial dynamics, gut microbiota interactions, and systemic inflammatory responses [17,21,38,39,40,41].Companion animal models demonstrated strong translational relevance due to their physiological similarity to humans, particularly in pancreatic cell composition and responses to diabetogenic stimuli [26,36,40].

Individual findings further highlighted notable interspecies variability: rodents showed strain-dependent differences in insulin secretion and glucose tolerance, whereas dogs and cats more closely reflected human patterns of IR and β-cell dysfunction [26,36,37]. Variation in microbiota composition across species influenced glucose metabolism, low-grade inflammation, and metabolic resilience, underscoring the importance of gut–metabolic interactions in DM progression [36,42,43].

Data synthesis revealed that although core insulin signaling pathways are evolutionarily conserved, species differ profoundly in β-cell regenerative capacity, mitochondrial efficiency, and microbiota-derived metabolic outputs [21,44]. These mechanistic distinctions are critical for selecting appropriate preclinical models and for ensuring accurate translational interpretation.

Limitations across studies included small sample sizes, heterogeneous experimental protocols, and incomplete reporting of molecular outcomes. Publication bias and underreporting of neutral findings may also distort overall interpretations [11,16,45,46]. Recognizing these sources of uncertainty is essential for critically evaluating evidence quality.

Despite these limitations, canine and feline models offer valuable opportunities for assessing antidiabetic therapies and conducting longitudinal studies on disease progression. Integrating molecular insights with clinical observations enhances the development of species-specific therapeutic strategies and supports the identification of predictive biomarkers [28,36,47]. These cellular and molecular distinctions form the mechanistic foundation for the interspecies differences in insulin secretion and sensitivity discussed in Section 3.3.

Therefore, the careful selection of animal models and the standardization of methodological approaches remain essential for generating robust and comparable translational data, ultimately strengthening interspecies research and future clinical applications.

### 3.3. Interspecies Differences in Insulin Response and Secretion

Interspecies differences in insulin response and secretion arise from physiological, genetic, and nutritional factors and are essential for interpreting Homeostatic Model Assessment (HOMA) indices in veterinary and translational medicine [31,43,48]. During the systematic selection process, 43 studies evaluating insulin dynamics in mammals were identified, of which nine met the inclusion criteria based on PRISMA guidelines. Two studies lacked quantitative glucose and insulin measurements under standardized conditions and were excluded for incomplete outcome reporting [14,29,43]. Ultimately, seven studies were included that provided reliable data on HOMA-derived indices, insulin response, and species-specific physiological characteristics.

Comparative evidence highlights reduced insulin sensitivity in cats, strongly associated with high-protein and high-fat diets, which influence their metabolic phenotype and predisposition to T2DM [14,25]. In dogs, structural and functional characteristics of pancreatic islets closely resemble those in humans, supporting their use as translational models for islet transplantation research and β-cell regenerative studies [14,25,31]. Clinical and biochemical assessments of diabetic cats show pronounced variation in insulin secretion, underscoring the need for standardized hormonal assays and uniform diagnostic protocols in veterinary endocrinology [31,49].

**Figure 3:**
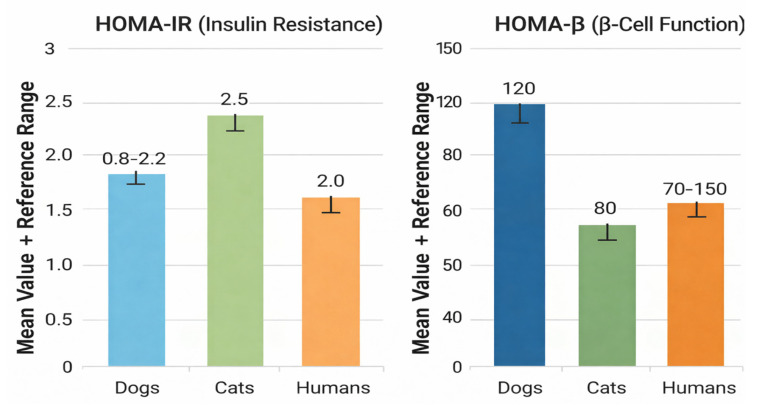
Comparative Assessment of HOMA-IR and HOMA-β Across Species.

Systematic integrative analyses support the use of HOMA indices for assessing β-cell function in both species, demonstrating distinct reference intervals and a strong correlation between HOMA-insulin resistance (HOMA-IR) and disease severity [29,42]. Studies validating HOMA-IR in cats confirm its predictive value for hyperinsulinemia, impaired glucose tolerance, and increased risk of T2DM [29,50]. Figure 3 illustrates interspecies differences in mean values and reference ranges for HOMA-IR and HOMA-β (β-cell function) in dogs, cats, and humans [29,31,42].

Figure 3 compares mean values and reference ranges of HOMA-IR and HOMA-β among dogs, cats, and humans, highlighting species-specific differences in insulin resistance and β-cell function.

Experimental studies in rodents with induced DM showed partial improvements in insulin sensitivity following bariatric interventions, but also considerable inter-individual variability, reflecting fundamental differences in metabolic plasticity between rodents and carnivores [29,43,45]. Comparative analyses of antidiabetic therapies further demonstrated species-dependent pharmacological responses, influenced by differences in metabolism and drug pharmacokinetics, supporting the need for tailored therapeutic approaches in humans, dogs, and cats [28,45].

Recent computational models integrating hybrid datasets and counterfactual analysis improved the accuracy of metabolic risk prediction, contributing to the development of advanced tools for early identification of IR [40,51,52,53].

### 3.4. The Role of the Microbiome and Environmental Factors in the Development of DM

The intestinal microbiome plays a pivotal role in regulating energy homeostasis and contributes significantly to the development and progression of DM in both humans and companion animals [14,38,48]. Evidence synthesized from eight eligible studies demonstrates that gut microbiota–metabolism interactions represent a conserved pathophysiological axis linking diet, inflammation, IR, and β-cell dysfunction across species [14,47,48].

**Figure 4:**
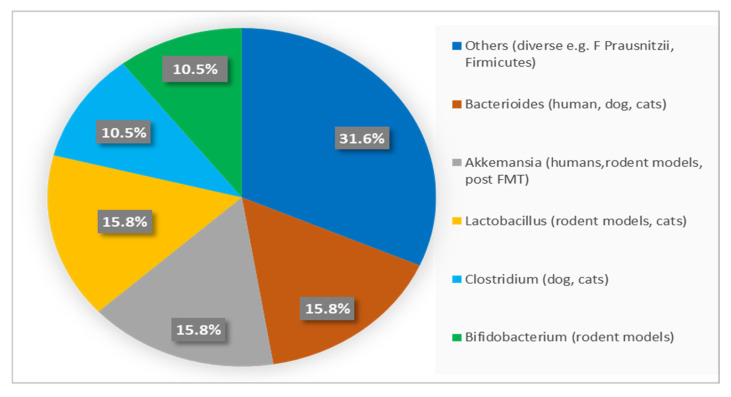
Frequency Distribution of Dominant Gut Bacterial Genera Across Species.

Rather than taxonomic composition alone, microbiome-associated metabolic functions appear to be the primary determinants of translational relevance. Reduced abundance of bacteria involved in short-chain fatty acid (SCFA) production—such as *Akkermansia muciniphila* and *Faecalibacterium prausnitzii*—has been consistently associated with increased intestinal permeability, low-grade systemic inflammation, and impaired insulin sensitivity [48,54]. Conversely, enrichment of SCFA-producing genera (e.g., *Bifidobacterium* spp. and *Lactobacillus* spp.) is linked to improved glucose homeostasis and preservation of β-cell function, as summarized in Table 2 and Figure 4 [14,55].

Although microbial taxa differ markedly among humans, dogs, cats, and rodents, key functional pathways—including SCFA synthesis, bile acid signaling, and immune modulation—are evolutionarily conserved. This functional convergence underpins the translational value of canine and feline microbiome studies for understanding metabolic regulation and inflammatory mechanisms relevant to human DM [43,54,55]. Accordingly, Figure 4 presents a semi-quantitative overview of dominant bacterial genera reported across species, facilitating interspecies comparison while minimizing bias related to methodological heterogeneity [14,48,55].

Percentages indicate the proportion of studies reporting each bacterial genus as dominant within species groups, providing a semi-quantitative comparison of interspecies microbiota patterns.

Dietary patterns and environmental exposures strongly modulate microbiome composition and metabolic outcomes. In domestic carnivores, protein- and fat-rich diets significantly influence gut microbial structure and insulin responsiveness. Cats consuming high-protein, high-fat diets exhibit reduced insulin sensitivity, contributing to their characteristic metabolic phenotype and increased susceptibility to T2DM [25]. Comparable diet–microbiome–metabolism interactions have been documented in rodent models and observational studies in companion animals, reinforcing their translational relevance for investigating metabolic dysregulation [47,48,56].

At a mechanistic level, microbiome-derived nanoparticles and extracellular vesicles (EVs) have emerged as important mediators of host–microbe communication, transporting bioactive molecules that influence inflammatory signaling, β-cell function, and glucose homeostasis [25,57]. Disruption of these pathways contributes to the interconnected processes of gut dysbiosis, obesity, and DM, commonly conceptualized as “diabesity,” in which pro-inflammatory microbial shifts enhance intestinal permeability and activate immune-mediated pathways that promote IR [38,48,58].

Microbiota-targeted interventions further highlight the clinical and translational potential of this axis. FMT has been shown to improve insulin sensitivity and partially restore β-cell function in experimental models of T2DM [48,49,59]. Additional molecular interventions affecting extracellular matrix remodeling and steroidogenesis-related enzymes have demonstrated beneficial effects on metabolic balance, although their clinical applicability requires further validation [48,54].

From a translational perspective, these findings emphasize that functional microbial pathways, rather than detailed taxonomic profiles, are most relevant for cross-species extrapolation [38,47,48,50,60]. Understanding these conserved mechanisms provides opportunities for developing personalized, microbiome-informed strategies aimed at preventing or delaying DM onset in both humans and animals. Recent cross-center initiatives, such as the Comparative Diabetes Database 2023 and the One Health Metabolic Consortium 2024 further support standardized data integration and enhance reproducibility in translational diabetes research [61,62].

### 3.5. Comparative Effects of Diet and Exercise on DM Across Species

Across species, dietary composition and physical activity significantly modulate metabolic control and influence disease progression in established DM. Numerous studies demonstrate that Western-type diets—high in refined carbohydrates, saturated fats, and ultra-processed foods—rapidly induce IR, chronic inflammation, and β-cell dysfunction in humans, rodents, and companion animals [63,64,65]. In contrast, traditional or developing-country diets, which typically include higher proportions of fiber, complex carbohydrates, and unsaturated fats, are associated with improved glycemic control, enhanced lipid metabolism, and reduced systemic inflammation [66,67,68].

Comparative findings indicate that diet-induced metabolic alterations are modulated by species-specific factors, including digestive efficiency, gut microbiota composition, and endocrine regulation. In dogs and cats, hypercaloric high-fat or high-protein commercial diets promote obesity and IR, whereas balanced, controlled diets restore metabolic flexibility and improve insulin sensitivity [25,48,55,69]. Rodent models fed hypercaloric diets exhibit hepatic steatosis and impaired glucose tolerance, reproducing early metabolic defects observed in human T2DM [64,68,69,70,71].

Physical activity interacts synergistically with dietary patterns to modulate glucose metabolism. Experimental and clinical data consistently show that regular aerobic or mixed exercise enhances insulin receptor sensitivity, increases mitochondrial efficiency, and reduces oxidative stress across species [34,72]. In dogs, moderate exercise regimens lower fasting glucose and triglyceride concentrations, while sedentary lifestyles are linked to increased obesity prevalence and DM complications [73,74]. Human studies further confirm that structured exercise programs—independent of caloric restriction—significantly improve β-cell responsiveness and reduce glycated hemoglobin (HbA1c) levels [75,76].

Lifestyle-related disparities between Western and developing regions also extend to companion animals. Dogs and cats in high-income countries frequently experience overnutrition, limited physical activity, and obesity-related DM. In contrast, animals in low-income or rural contexts show lower obesity rates but greater exposure to infectious comorbidities that exacerbate metabolic stress [47,77,78,79,80,81]. These observations highlight the need to contextualize metabolic outcomes within environmental and sociocultural frameworks.

Integrating dietary and physical activity parameters into comparative DM research increases translational validity and enables the identification of modifiable lifestyle risk factors that influence disease susceptibility and therapeutic outcomes across species. Future studies should adopt standardized, cross-species protocols to elucidate how nutrition and exercise interact with genetics and the microbiome to shape species-specific metabolic phenotypes [80,81,82,83].

### 3.6. Autonomic Nervous System (ANS) and Species-Specific Neuroendocrine Regulation

The ANS is a key regulator of glucose homeostasis, coordinating central and peripheral mechanisms that control insulin secretion, hepatic glucose production, and peripheral glucose uptake. Dysregulation of the sympathetic–parasympathetic balance contributes to the onset and progression of DM, influencing IR, β-cell survival, and vascular function [81,82,83,84]. Comparative studies demonstrate that interspecies differences in ANS activity significantly affect metabolic outcomes and the development of DM complications [85,86,87].

In rodent models, increased sympathetic activation and reduced parasympathetic tone often precede the onset of hyperglycemia, indicating an early neurogenic component in experimental DM. Dogs exhibit marked autonomic variability, with parasympathetic withdrawal and sympathetic overactivation associated with gastrointestinal and cardiovascular complications similar to those observed in human diabetic autonomic neuropathy. In cats, reduced vagal activity has been linked to impaired insulin sensitivity and delayed gastric emptying, suggesting a species-specific neuroendocrine pattern that parallels human T2DM [25,27,47,86].

At the cellular level, catecholamine- and acetylcholine-mediated signaling modulates β-cell secretion, adipose tissue metabolism, and whole-body energy expenditure. Chronic sympathetic hyperactivation increases oxidative stress and mitochondrial dysfunction—mechanisms consistently reported across animal models and human studies [88,89]. Metabolomic analyses further indicate that autonomic imbalance influences inflammatory cytokine expression and gut–brain axis communication, reinforcing the interplay between neural regulation and metabolic disease progression.

From a translational standpoint, the ANS represents a critical but underexplored interface between neuroendocrine and metabolic pathways. Quantitative assessments such as heart rate variability (HRV), baroreflex sensitivity, and sympathetic skin response in animal models provide measurable correlates of autonomic function that can bridge veterinary and human research. Incorporating autonomic biomarkers into experimental designs may improve early detection of DM neuropathy and enhance the predictive value of translational models for neuroendocrine complications of DM.

### 3.7. Species-Specific Complications of DM

DM is associated with a wide range of organ-specific complications—including retinopathy, nephropathy, neuropathy, and cardiovascular disorders—that differ markedly across species [1,6,61]. The systematic evaluation of included studies revealed a moderate risk of bias in some observational studies due to incomplete reporting, particularly concerning neuropathic and cardiovascular outcomes in dogs and cats [25,27,64]. In contrast, controlled experimental studies, especially those conducted in rodent models, demonstrated a low risk of bias owing to rigorous control of variables and standardized outcome assessments [42,65].

The certainty of evidence varied across outcomes. Human studies addressing retinopathy, nephropathy, and neuropathy exhibited high certainty because of large cohort sizes and long-term follow-up [65,66]. Evidence from veterinary studies demonstrated moderate certainty, limited by smaller sample sizes, variability in clinical management, and underreporting of subclinical complications [25,57]. These findings highlight the need for standardized diagnostic criteria and longitudinal monitoring in companion animals.

Overall, humans exhibit the highest prevalence of DM complications, followed by dogs and cats. Retinopathy affects 30–40% of human patients, compared with 5–10% of dogs and <5% of cats [25,26,42,67,82]. Nephropathy and neuropathy follow a similar gradient, while cardiovascular complications are rare in companion animals but remain prominent in humans [25,26,27]. Table 3 summarizes the estimated prevalence of major DM complications across species, reflecting differences in lifespan, metabolic adaptations, and clinical management practices.

Evidence limitations include reporting inconsistencies, underrepresentation of feline-specific data, and heterogeneous diagnostic approaches across studies. Additionally, limited longitudinal evidence restricts the ability to quantify the progression and severity of complications in dogs and cats [57,59,68]. Review-level limitations include the restriction to English-language publications, potential selection bias toward studies reporting quantifiable outcomes, and the exclusion of gray literature, which may underestimate rare conditions.

Despite these constraints, the present synthesis provides a transparent, PRISMA-aligned overview of interspecies differences in DM complications. Implications for clinical practice and translational research include the need for standardized veterinary guidelines for screening DM complications, improved reporting quality in clinical studies, and enhanced integration of animal and human evidence. Targeted interventions should account for species-specific susceptibilities—particularly regarding nephropathy and neuropathy—to optimize management strategies and reduce morbidity.

### 3.8. Translational Relevance of Animal Models in DM Research

Building on the general translational considerations discussed above, this section focuses on the translational relevance of specific animal models used in diabetes research.

Translational medicine bridges experimental discovery and clinical application by employing animal models to replicate metabolic, molecular, and immunologic processes relevant to human DM [15,18,31,32,33]. Canine and feline models provide realistic representations of spontaneous DM, exhibiting clinical, hormonal, and histopathologic features comparable to human T1DM and T2DM, respectively [14,31]. These similarities support their use as translational platforms that enable the transition from experimental observations to predictive clinical interventions. Although animal models cannot fully replicate human disease complexity, studies in dogs and cats have yielded key insights—such as the success of islet transplantation in canine DM and mechanisms of obesity-associated IR in feline models—that have directly informed human DM research [25,28,29].

The integration of computational modeling has added significant value to translational research. Mathematical simulations of glucose–insulin dynamics and hormonal feedback loops offer high predictive accuracy and facilitate hypothesis testing without invasive procedures [14,18,31]. Molecular systems approach—including transcriptomics and proteomics—have revealed conserved signatures across species, emphasizing evolutionary preservation of pathways implicated in β-cell dysfunction [15,17,32]. However, interspecies differences remain notable. Comparative mitochondrial studies highlight distinct metabolic efficiencies that may influence cardiovascular outcomes and responsiveness to therapy [21,32].

According to the European Society for Translational Medicine, effective translation requires a continuous, bidirectional exchange between fundamental discovery and clinical validation [9,21,32]. Nonetheless, reproducibility challenges continue to limit translational robustness. Variability in experimental protocols and inherent species differences contribute to inconsistent outcomes. Murine models, for example, despite their utility in genetic studies, show substantial structural differences in pancreatic islets and divergent immune responses relative to humans, limiting their predictive accuracy [23,32,69].

A comparative analysis of major DM animal models, summarized in Table 1, illustrates substantial variation in translational fidelity. Dogs demonstrate the highest correlation with human T1DM (82%), followed by cats (76%), which parallel human T2DM pathophysiology [16,22]. Pigs exhibit moderate concordance (65%) due to similarities in lipid metabolism and pancreatic architecture, while rodents show lower correlation (54%), reflecting pronounced metabolic and immunologic divergence [16,17,32]. Non-human primates, particularly Cynomolgus and Rhesus macaques, show the highest molecular similarity (88%) and are widely used for evaluating incretin therapies and islet transplantation [23,28,70].

Differences highlighted in Table 1 confirm that canine and feline models offer superior predictive accuracy compared with rodents, largely due to natural disease progression and endocrine similarities with humans. However, factors such as genetic variability, high maintenance costs, and heterogeneous housing conditions may introduce methodological biases that limit reproducibility. Overall, the certainty of evidence supporting these models is moderate to high, strengthened by consistency across both experimental and clinical studies [22,33].

A further limitation concerns reporting bias, as studies tend to favor publication of positive results while underreporting neutral or negative findings, potentially distorting evaluations of model efficiency [11,33]. Additionally, the scarcity of long-term longitudinal studies restricts understanding of natural disease trajectories in companion animals, reducing confidence in meta-analytic inferences [16,33].

Emerging research expands the translational scope of DM models by exploring neuroendocrine mechanisms—such as oxytocin-mediated regulation of feeding behavior and metabolism in dogs—which show promising relevance for comparative physiology [16,33,47]. Studies on aging and oxidative stress similarly contribute to identifying mechanistic overlaps between metabolic decline and diabetic pathophysiology [47,57].

Despite species-specific differences in toxicological responses and metabolic risk assessment [20,57], phase I/II veterinary clinical trials, including those in metabolic oncology, demonstrate the practical translational value of companion animal models by providing directly applicable insights for human therapeutics [33,57,70].

Overall, translational research in DM relies on an iterative feedback process linking animal modeling, computational analyses, and clinical observations. Strengthening this continuum requires interdisciplinary collaboration, standardization of experimental protocols, and systematic integration of multi-omic datasets to ensure predictive validity and uphold ethical research standards [9,15,21,65].

### 3.9. Future Directions for Research and Standardization of Models

Despite substantial progress in comparative endocrinology, research on DM across species continues to be challenged by methodological heterogeneity and biological variability [38,39,52]. Variations in experimental design, diet composition, and microbiome characterization reduce cross-study comparability and weaken external validity [18,71,72,77]. Harmonizing methodological frameworks and integrating multi-omic datasets therefore remain critical priorities for improving reproducibility and translational predictability.

The studies analyzed encompass a broad range of experimental and clinical models—including murine, canine, feline, porcine, and human cohorts—addressing key domains such as microbiota–metabolism interactions, IR, and gestational glucose regulation [51,73,74]. Evidence consistently shows that species-specific microbiota profiles influence insulin sensitivity, systemic inflammation, and glucose homeostasis. Microbiota-targeted interventions, including FMT, have demonstrated reproducible metabolic benefits across models, reinforcing the need for standardized microbiome methodologies and harmonized sampling protocols [36,48,70,71,72,77]. Similarly, studies on gestational diabetes strengthen associations between glucose dysregulation and adverse pregnancy outcomes in large clinical populations [70,77,78].

Although all included studies used validated metabolic and microbiological tests, variability in sample size, species selection, and housing conditions introduces sources of bias [77,78]. Environmental factors significantly modulate the murine microbiome, complicating extrapolation to companion animals [68,69,70]. While reporting bias was generally low, several studies lacked explicit randomization or blinding, which may influence outcome interpretation [36,67,68,73]. The limited availability of negative or null results also raises concerns about publication bias, particularly in studies evaluating microbiota-based interventions [38,51,68,74].

Heterogeneity in interspecies metabolic responses reflects inconsistent protocols, divergent diet composition, and variation in microbiome sequencing methods (metagenomics vs. 16S rRNA) [54,67,75]. Standardizing sampling strategies, analytical techniques, and reporting frameworks is essential for improving reproducibility and ensuring external validity across species [38,51,70,71,72,77,79].

According to the GRADE framework, overall certainty of evidence linking microbiota composition to metabolic regulation is moderate to high in rodent and human studies, but low to moderate for canine and feline studies due to smaller sample sizes and limited validation [20,36,72]. Evidence supporting microbiota-based therapeutic interventions, including FMT, is moderate, whereas research on gestational DM displays high certainty and reproducibility across clinical cohorts [66,67,68,69,70,71,77]. Nonetheless, gaps remain due to small sample sizes, species-specific physiology, and the scarcity of longitudinal studies in companion animals [66,67,68].

Cross-species integration of molecular, metabolic, and environmental parameters is essential for improving translational potential [72,73,80,81,82]. Harmonization of protocols across laboratories will strengthen the translational impact of comparative metabolic studies. Multicenter collaborations, data-sharing initiatives, and unified analytical pipelines are crucial for validating biomarkers and therapeutic targets [66,72,73,81,83].

These principles are summarized in Table 4, which outlines priority directions for standardizing and integrating DM research across species.

**Figure 5:**
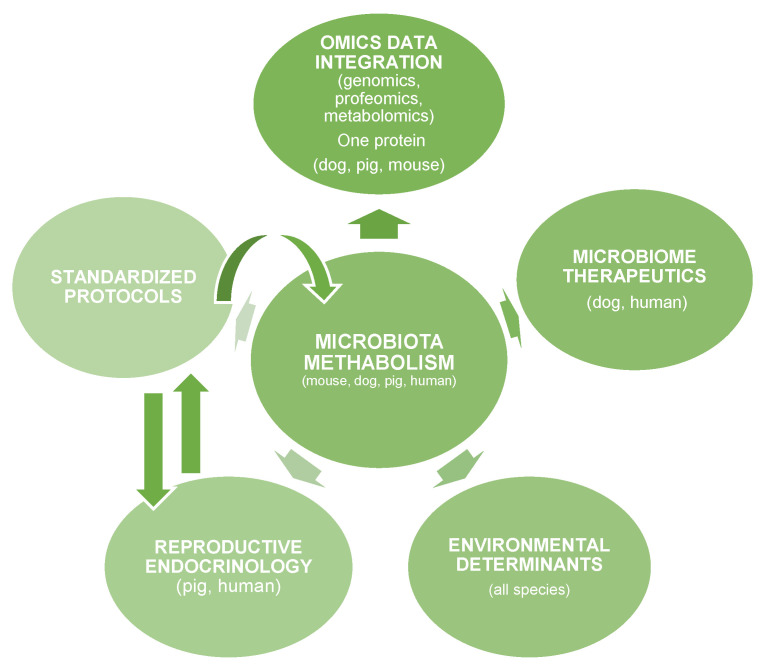
The Translational Microbiota-Metabolism Research Ecosystem.

Emerging research domains such as reproductive endocrinology, environmental determinants, microbiome therapeutics, and omics data integration are further illustrated in Figure 5, which provides a conceptual overview of the translational microbiota–metabolism ecosystem.

Figure 5 presents a schematic overview of the key interconnected domains linking microbiota metabolism with translational diabetes research across species, within a “One Health” framework.

Owner lifestyle and environmental context significantly influence diabetic outcomes in companion animals. Western feeding patterns and reduced physical activity are associated with obesity, dyslipidemia, and chronic inflammation, whereas non-Western settings show lower obesity prevalence but higher rates of infectious comorbidities that exacerbate metabolic stress [26,30,43,54]. These findings emphasize the interdependence between owner behavior and companion-animal metabolic health and reflect lifestyle-related risks shared with human DM.

Although evidence regarding DM in companion animals remains fragmented, it is increasingly promising [75,78]. Methodological heterogeneity, moderate bias, and species variability limit generalizability [73,74,76]. However, integrating standardized methodologies and multi-omic approaches can substantially enhance the certainty and translational relevance of future findings [66,78]. Ultimately, a harmonized research framework—supported by cross-species collaboration and reproducible metabolic analyses—has the potential to bridge veterinary and human diabetes research through robust interspecies translational perspectives [74,75,76,78].

### 3.10. Limits and Challenges in the Study of Interspecies Variability

Research on interspecies variability in DM is constrained by significant methodological and biological challenges across rodent, companion-animal, and large-animal models, with major implications for reproducibility and translational applicability [16,79,80,81]. One of the central limitations is data heterogeneity, driven by differences in diabetes induction methods, diagnostic criteria, and metabolic markers used across studies [23,79,80]. Experimental models of T1DM and T2DM exhibit distinct immunological pathways and treatment responses, reducing opportunities for direct data integration [79,80,81].

Species differences shape the natural history of DM. While rodents typically develop experimentally induced hyperglycemia, dogs and cats present spontaneous disease forms strongly influenced by endocrine factors and chronic stress [78,82,83]. This divergence contributes to variability in insulin sensitivity and β-cell function reported across studies [78,82]. Limited translatability remains a major obstacle, as no animal model fully reproduces the complex pathophysiology of human DM, despite shared core mechanisms [23,78]. Structural differences in pancreatic islets between rodents and humans affect inflammatory and oxidative-stress responses [43,81,83], and animal models often show an accelerated disease progression compared to the gradual course observed in humans [78]. These discrepancies are particularly evident in preclinical cellular and pharmacological therapy studies, where human clinical success rates lag behind those observed in laboratory models [31,43].

Companion animals provide valuable models for spontaneous DM, yet dogs and cats remain underrepresented in comparative research, limiting generalizability [80,81,82,83]. The scarcity of cross-species studies reduces understanding of hormonal, metabolic, and behavioral determinants of disease progression [83,84]. Rodent findings are often extrapolated without adequate adjustment for species-specific physiology [83,84,85]. Additional confounding factors—including diet, age, sex, genetic background, and environmental stress—further influence insulin sensitivity and glycemic regulation [83,84,85]. Variations in cortisol secretion and neuroendocrine pathways generate divergent metabolic responses across species [81,84,85]. Murine models, in particular, are sensitive to microclimatic variations, introducing metabolic fluctuations that compromise reproducibility [72,85].

Differences in experimental protocols for DM induction, complication assessment, and metabolic marker quantification continue to hinder comparability across laboratories [78,85]. Integration of genomic, proteomic, and metabolomic technologies remains limited, despite their potential to identify predictive biomarkers and support precision medicine approaches [8,84].

Systematic evaluations reveal inconsistencies in selection bias, reporting bias, and methodological quality [43,81]. The absence of randomization and blinding in some studies increases the risk of systematic error [51,86,87,88], while selective reporting of positive outcomes contributes to publication bias [43,81,89]. Consequently, certainty of evidence in preclinical animal studies remains moderate [23,84].

Addressing these limitations requires integrating molecular, metabolic, and clinical parameters across species [23,86,87,88]. Large-animal models such as pigs and companion animals such as dogs can provide more relevant data for human physiology, although high costs and genetic diversity pose additional challenges [43,86,89]. Advanced molecular imaging and sequencing technologies support interspecies validation of mechanistic findings and promote the development of personalized interventions [23,51,84]. Strengthening collaboration between veterinary and human medicine is essential for increasing the translational reliability of animal models, as summarized in Table 5 [23,84,86,89].

## 4. Conclusions

This systematic review demonstrates that interspecies variability in DM provides essential insights for translational medicine. Canine and feline models most closely reflect the clinical, metabolic, and pathophysiological characteristics of human DM, offering substantial predictive value for mechanistic investigations and therapeutic development. Although core pathways underlying insulin signaling and β-cell dysfunction are evolutionarily conserved, species-specific differences continue to influence disease expression, progression, and treatment response.

Persistent heterogeneity in study design, diagnostic criteria, and metabolic assessment limits reproducibility and reduces the overall certainty of available evidence. The implementation of standardized methodologies, harmonized reporting frameworks, and integrated molecular–clinical approaches across species is therefore critical to improving translational reliability.

Future research should prioritize multi-omic, cross-species analytical strategies that connect experimental and clinical endocrinology. Strengthening the OH translational framework—through standardized data sharing, ethical refinement, and cross-center collaboration—is essential for bridging gaps between rodent models and spontaneous diabetes in companion animals. Such integration will enhance predictive validity and reinforce the continuum from mechanistic discovery to clinically meaningful therapeutic applications.

## Figures and Tables

**Table 1:** Translational relevance and limitations of DM models across species [15,16,17,18,21,27,31,32,37,38].

Species/Model	Type of DM Modeled	Key Pathophysiological Features	Translational Relevance	Major Limitations
Rodent (mouse, rat)	T1DM/T2DM (induced, genetic)	Chemically induced β-cell loss; genetic obesity and insulin resistance; rapid disease onset	Mechanistic and molecular studies; pathway discovery	Limited chronicity; divergent islet architecture; reduced predictive value for long-term outcomes
Dog	T1DM (spontaneous, induced)	Immune-mediated β-cell destruction; complete insulin dependence; chronic hyperglycemia	High translational relevance for human T1DM; islet transplantation and immunotherapy models	Cost; genetic heterogeneity; limited availability
Cat	T2DM (spontaneous, diet-induced)	Obesity-associated insulin resistance; progressive β-cell dysfunction	High relevance for early human T2DM and metabolic syndrome	Late diagnosis; dietary variability; limited longitudinal data
Pig	T2DM (diet-induced)	Similar pancreatic anatomy and lipid metabolism to humans	Moderate translational value for metabolic and cardiovascular studies	High cost; housing and handling constraints
Non-human primate	T2DM (spontaneous or diet-induced)	β-cell structure, incretin response, immune regulation closely resemble humans	Very high translational relevance for advanced preclinical studies	Ethical concerns; high financial and regulatory burden
Human (reference)	T1DM/T2DM	Full clinical spectrum; chronic disease progression	Reference standard for translational validation	Heterogeneity; ethical and practical constraints

Note: Comparative overview of major animal models used in diabetes research, highlighting the type of diabetes modeled, key pathophysiological features, translational relevance, and principal limitations across species.

**Table 2:** Species-specific gut microbiota and metabolic implications for DM [14,38,47,48,50,51,53].

Species	Experimental Condition/Group	Dominant Gut Bacterial Phyla/Genera	Metabolic Features	Implications for Diabetes
Humans		*Firmicutes*, *Bacteroidetes*, *Akkermansia muciniphila*, *Faecalibacterium prausnitzii.*	Balanced carbohydrate fermentation, short-chain fatty acid (SCFA) production, anti-inflammatory effect.	Reduced abundance of *A. muciniphila* and *F. prausnitzii* linked to IR and low-grade inflammation.
Dogs		*Lactobacillus*, *Clostridium*, *Bacteroides*, *Prevotella.*	Efficient carbohydrate metabolism.	Dysbiosis associated with IR.
Cats		*Bacteroides*, *Enterococcus*, *Clostridium*, *Fusobacterium.*	Protein- and fat-adapted metabolism.	Reduced Lactobacillus populations and high *Bacteroides* levels contribute to lower insulin sensitivity.
Rodent models	STZ-induced diabetes	*Lactobacillus*, *Bifidobacterium*, *Desulfovibrio*, *Akkermansia.*	Sensitive to dietary modulation; rapid microbiota turnover.	High-fat diets induce dysbiosis, leading to inflammation and impaired glucose tolerance.
Dogs	Post–fecal microbiota transplantation (FMT)	Increasing of *Akkermansia* and *Bifidobacterium abundance.*	Restoration of gut barrier integrity and improved insulin sensitivity.	Partial reversal of IR and β-cell dysfunction observed after FMT.

Note: Comparative overview of dominant gut bacterial taxa, metabolic features, and implications for DM across species and experimental conditions (including FMT).

**Table 3:** Comparative prevalence of DM complications across species (retinopathy, nephropathy, neuropathy, and cardiovascular disorders) [25,26,27,42,56,57,58,59].

Species	Retinopathy (%)	Nephropathy (%)	Neuropathy (%)	Cardiovascular Complications (%)
Humans	30–40	20–30	30–50	25–35
Dogs	5–10	10–15	15	10–15
Cats	<5	3–7	10	<5

Note: Estimated prevalence ranges of major diabetic complications across species, highlighting interspecies differences in microvascular and macrovascular outcomes.

**Table 4:** Future directions for standardization and translational integration in DM animal models [68,70,71,72,73,77].

Focus Area	Objective	Species Involved	Translational Relevance
Microbiota–metabolism axis	Define interspecies microbial signatures influencing glucose homeostasis	Mouse, dog, cat, human	High–identifies conserved microbial pathways
Omics data integration	Combine genomic, proteomic, and metabolomic datasets across species	Dog, pig, mouse	Very high–enhances predictive biomarkers
Standardized protocols	Harmonize diet, housing, and sampling protocols in translational studies	Mouse, cat, dog	High–improves reproducibility
Microbiome therapeutics	Evaluate FMT as a treatment model	Dog, human	High–demonstrates causal metabolic reversal
Environmental determinants	Identify and control external risk factors influencing DM development	All species	Moderate–reduces study bias
Reproductive endocrinology	Investigate gestational DM as a translational model	Pig, human	Moderate–relevant to metabolic adaptation

Note: Legend—Conclusions emphasize conserved microbial functions rather than taxonomic differences, supporting translational interpretation of microbiome data across species [43,54,55,61,84,85,86,87].

**Table 5:** Limitations and Determinants of Interspecies Variability in DM Models [78,79,80,84,85].

Category	Description	Impact on Translation
Heterogeneity of Methods	Induction of diabetes through different mechanisms (chemical, genetic, spontaneous)	Decreases comparability between studies
Pathophysiological Differences	Divergences in pancreatic islet structure and inflammatory response	Limits extrapolation to humans
Confounding Factors	Diet, sex, age, genetics, stress	Introduces uncontrolled variations
Underrepresented Subjects	Lack of comparative studies on companion animals	Reduces the generalization of conclusions
Low Standardization	Variable protocols for metabolic testing and complication assessment	Affects reproducibility
Methodological Bias	Lack of randomization and reporting of negative results	Increases the risk of erroneous conclusions

Note: Determinants of interspecies variability in diabetes models [78,79,80,84,85].

## Data Availability

All data generated or analyzed during this study are included in this published article and its Supplementary Material.

## Supplementary Materials

The following supporting information can be downloaded at: https://www.mdpi.com/article/10.3390/life16010064/s1, PRISMA Checklist.
